# Diagnostic armamentarium of infectious keratitis: A comprehensive review

**DOI:** 10.1016/j.jtos.2021.11.003

**Published:** 2022-01

**Authors:** Darren S.J. Ting, Bhavesh P. Gopal, Rashmi Deshmukh, Gerami D. Seitzman, Dalia G. Said, Harminder S. Dua

**Affiliations:** a Academic Ophthalmology, School of Medicine, University of Nottingham, Nottingham, UK; b Department of Ophthalmology, Queen's Medical Centre, Nottingham, UK; c Department of Ophthalmology, L V Prasad Eye Institute, Hyderabad, India; d Francis I. Proctor Research Foundation, University of California, San Francisco, USA

**Keywords:** Artificial intelligence, Confocal microscopy, Corneal infection, Corneal ulcer, Diagnosis, Mass spectrometry, Next-generation sequencing, Polymerase chain reaction

## Abstract

Infectious keratitis (IK) represents the leading cause of corneal blindness worldwide, particularly in developing countries. A good outcome of IK is contingent upon timely and accurate diagnosis followed by appropriate interventions. Currently, IK is primarily diagnosed on clinical grounds supplemented by microbiological investigations such as microscopic examination with stains, and culture and sensitivity testing. Although this is the most widely accepted practice adopted in most regions, such an approach is challenged by several factors, including indistinguishable clinical features shared among different causative organisms, polymicrobial infection, long diagnostic turnaround time, and variably low culture positivity rate. In this review, we aim to provide a comprehensive overview of the current diagnostic armamentarium of IK, encompassing conventional microbiological investigations, molecular diagnostics (including polymerase chain reaction and mass spectrometry), and imaging modalities (including anterior segment optical coherence tomography and in vivo confocal microscopy). We also highlight the potential roles of emerging technologies such as next-generation sequencing, artificial intelligence-assisted platforms. and tele-medicine in shaping the future diagnostic landscape of IK.

## 1 Introduction

Infectious keratitis (IK) represents the leading cause of corneal blindness worldwide. According to the latest report published by the World Health Organization (WHO), IK has affected around 6 million population globally, particularly in under-resourced countries, and is estimated to account for an ongoing 1.5–2.0 million monocular blindness per year [1]. In addition, a US study has reported that approximately $175 million dollars were spent on IK annually, highlighting its significant economic burden on the healthcare system [2]. However, the global health, economic and societal impact of IK are likely to be underreported and underestimated as the majority of IK cases occur in middle- and low-income countries [3].

Successful management of IK is dependent on timely and accurate diagnosis followed by appropriate interventions. In principle, clinical diagnosis of any type of infection relies on a systematic synthesis of information gleaned from clinical history (with particular attention to important positive and negative risk factors), clinical examination, and microbiological investigations [4]. The same principle is applied to the diagnostic approach to IK, considering that IK can be caused by a wide range of organisms, including bacteria, fungi, viruses, parasites, and polymicrobial infection, which frequently pose significant diagnostic and therapeutic challenges [[5], [6], [7]].

IK is primarily diagnosed on clinical grounds supplemented by microbiological investigations such as microscopic examination with staining and culture and sensitivity testing. This is the most widely accepted practice adopted in most countries, or at least in places where resources and microbiology facility are available. Clinical history can often shed light on the possible underlying causative organisms of IK. For instance, contact lens wear is more commonly associated with *Pseudomonas aeruginosa* and Acanthamoeba keratitis [[8], [9], [10]], whereas corneal trauma caused by vegetative matter is more likely linked to fungal infection [11]. Characteristic clinical features such as dendritic-shaped ulcers (in herpetic keratitis) [12], feathery borders and satellite lesions (in fungal keratitis) [[13], [14], [15]], and perineural/ring infiltrates (in Acanthamoeba keratitis) [13,16], may sometimes provide additional clues to the underlying cause.

**Fig. 1:**
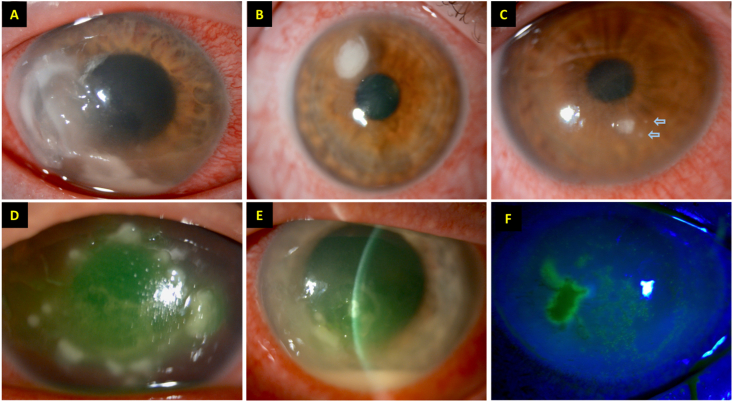
Slit-lamp photographs demonstrating different types of infectious keratitis (IK), highlighting the clinical challenges of diagnosing IK without any microbiological investigation. **(A**–**C)** Three separate cases of IK caused by *Pseudomonas aeruginosa*. Note the difference in the severity and clinical appearance among the three cases. The two “satellite lesions” (blue arrows) depicted in **(C)** image may give a false impression of fungal keratitis. **(D)** A case of IK caused by *Staphylococcus aureus* two days after corneal cross-linking treatment. **(E**–**F)** A case of polymicrobial IK, caused by *S. aureus* and herpes simplex keratitis, in a patient with atopic keratoconjunctivitis. (For interpretation of the references to colour in this figure legend, the reader is referred to the Web version of this article.)

However, obtaining an accurate clinical diagnosis of IK in a real-world setting is often challenging. This is primarily attributed to several factors, including indistinguishable clinical features shared among different causative organisms, polymicrobial infection, variably low culture positivity rate, and long turnaround time for diagnostic testing (Fig. 1A–F) [13,15,[17], [18], [19]]. In view of these limitations, a number of adjuvant imaging techniques and molecular diagnostic tools have been developed and utilized to improve the speed and accuracy of IK diagnosis in recent years.

In this review, we aim to provide a comprehensive overview of the current diagnostic armamentarium of IK, encompassing a range of microbiological investigations, corneal imaging modalities and molecular diagnostics, and discuss the strengths and limitations of each technique. In addition, we highlight the potential of emerging technologies such as clinical metagenomic next-generation sequencing, artificial intelligence-assisted platforms and tele-medicine in shaping the future diagnostic landscape of IK.

## 2 Current diagnostic armamentarium

A range of diagnostic modalities, including microbiological investigations, corneal imaging and molecular diagnostics, are available to aid the diagnosis of IK. In this section, we provide a succinct overview of the fundamental principles, clinical utility, diagnostic performance, strengths, and limitations of each diagnostic technique.

### 2.1 Conventional microbiological investigations

Microbiological investigations such as corneal scraping for culture and sensitivity testing remains the gold standard in diagnosing IK. Based on corneal photographs, Dalmon et al. [20] demonstrated that cornea specialists could only distinguish bacterial keratitis from fungal keratitis in 66% of the cases. Another study by Yildiz et al. [21] similarly observed that typical features of fungal keratitis such as satellite lesions or feathery infiltrates were only present in ∼30% of all cases, underscoring the importance of microbiological work-up during the diagnostic process of IK. In addition, corneal culture allows for determination of antimicrobial susceptibility and resistance, which is invaluable in guiding the choice of antimicrobial treatment in non-responsive IK cases.

#### 2.1.1 Corneal scraping

According to the American Academy of Ophthalmology (AAO) guidelines for managing bacterial keratitis, corneal scraping for microscopy, staining and culture is recommended in a number of clinical circumstances, including: (1) central, large corneal ulcer with or without stromal involvement; (2) chronic infection or cases refractory to ongoing treatment; (3) previous history of corneal surgeries; (4) atypical clinical features; and (5) multifocal corneal infiltrates [22]. Corneal scraping is optional for small peripheral corneal ulcers with limited stromal involvement. Samples for microbiological investigations must be obtained preferably before the onset of antimicrobial treatments as pre-treatment has been shown to affect the isolation rates of causative organisms [23,24].

Corneal scraping is generally performed under topical anesthesia at the slit lamp. In principle, loose mucus or debris is removed before scraping to increase the chances of isolating the causative organisms. Scraping should be performed at the base, the leading edges, or the most active site of the ulcer for maximal yield rate. The choice of topical anesthesia, scraping technique and instruments used have been shown to influence the culture yield of causative microorganisms [[25], [26], [27], [28]]. Proxymetacaine 0.5% exhibits less antibacterial effect than tetracaine 1% and oxybuprocaine 0.4% and hence the former is preferred [26]. A wide range of instruments have been used for corneal scraping, including non-metallic instruments such as cotton-tipped applicator and calcium alginate swab, and metallic instruments like Kimura spatula, surgical blade, and needle [27,28]. In general, swabs have higher absorbent properties giving higher culture positive rates, though the scrapes obtained are more superficial. Consequently, swabs are also considered safer to use in thin cornea with risk of perforation. Several studies have shown that calcium alginate swab moistened with trypticase soy broth had a higher recovery rate of organisms in IK compared to spatula [27]. Dry cotton tipped applicator has been shown to yield a significantly higher positive culture rate than surgical blade [28]. However, the type of swab can potentially affect the yield of particular organisms. For instance, calcium alginate and tannins found in wood swabs can be inhibitory to polymerase chain reaction (PCR) yield [29]. In addition, the culture yield based on cotton swabs is influenced by the swab material, the organisms, and the surface characteristics [30]. On the other hand, needle, blade or spatula can facilitate corneal sampling from the deeper corneal layers, and debridement of the infected cornea enhances the penetration of antimicrobial drugs.

In light of the relatively low culture yield rate (37.7%) observed in our recent Nottingham Infectious Keratitis Study [19], we have recently shifted from using needles or blades to using flocked swab (Appleton Woods Ltd, Birmingham) for obtaining corneal samples. The tip of the flocked swab is coated with perpendicularly sprayed on nylon fibres, which has been shown to increase the uptake and release of analytes (e.g. microbes) and enhance the culture yield rate [31,32].

#### 2.1.2 Microscopic examination with staining

Following corneal scraping, the obtained material is directly transferred onto the slide for direct microscopy and staining. Compared to culture, it has the advantage of providing microbiological results in a very short turnaround time, which is crucial in the management of IK. Smears are usually performed on two slides; one is for Gram staining for bacteria and another one is for Giemsa staining or potassium hydroxide (KOH) wet mount for fungi. Apart from these staining methods, other special stains such as calcofluor white (CFW), Gomori-methenamine-silver (GMS), lactophenol cotton blue (LPCB), Ziehl-Neelsen (ZN), and Kinyoun stains, amongst others, have been used (Table 1) [17,33,34].

**Table 1:** A summary of staining methods commonly used for identification of causative organisms of infectious keratitis.

Staining	B	F	A	N	MB	MS	C	O
Gram	Y	Y	Y	Y	–	Y	Y	–
Giemsa	Y	Y	Y	Y	–	Y	Y	Y^a^
10% KOH	–	Y	Y	Y	–	Y	–	–
Calcofluor white	–	Y	Y	–	–	Y	–	–
Ziehl Neelsen	–	–	Y	Y	Y	Y	–	–
Kinyoun	–	–	–	Y	Y	Y	–	–
Acridine orange	Y	Y	Y	Y	Y	Y	–	–
GMS	–	Y	–	–	–	Y	–	–
LPCB	–	Y	Y	–	–	–	–	–
IKI-H_2_SO_4_	–	–	–	–	–	–	–	Y^b^

B = Bacteria; F = Fungi; A = Acanthamoeba; N = Nocardia; MB = Mycobacterium; MS = Microsporidia; C = Chlamydia; O = Others; KOH = Potassium hydroxide; GMS = Gomori-Methanamine-Silver; LPCB = Lactophenol cotton blue; IKI-H_2_SO_4_ = Potassium iodide-sulfuric acid.

a Used to identify viral inclusion bodies in herpetic keratitis.

b Used to identify Pythium species.

In general, larger or more severe ulcers are associated with a higher smear and culture positivity rate, owing to a higher amount of bacterial bioburden and available samples for scraping and testing [10,33]. Gram staining is the most common staining method used to identify and classify bacteria in IK, with an overall sensitivity of 36–100% [23,33,35,36]. However, the results have been shown to vary significantly between laboratories/institutes due to variable interpretation accuracies/errors [37], highlighting the need for correlating the findings with the culture results. Gram-positive bacteria contain a thick peptidoglycan layer in their cell wall, which retains the crystal violet dye and appear violet on Gram staining. In contrast, Gram-negative bacteria only possess a thin peptidoglycan layer and hence are unable to retain the crystal violet dye during the discoloration stage by ethanol. They are subsequently counterstained pink by safranin. *Nocardia* spp. appear as weakly stained Gram-positive, beaded filaments with branching, whereas unstained or partially stained bacilli indicate a possible presence of *Mycobacterium* spp. [33,34]. Though not commonly used, acridine orange demonstrated comparable or higher detection rate than Gram staining, particularly in mild IK cases [38,39].

Giemsa staining and 10% KOH wet mounts are commonly used to identify fungi in IK cases, with an overall sensitivity of 40–85% and 81–99%, respectively [33,[40], [41], [42], [43]]. Their sensitivity in detecting fungi can be further improved with the addition of CFW stain [40]. In addition, Giemsa stain helps identify chlamydial and viral inclusion bodies as well as *Acanthamoeba* cysts and trophozoites [44]. KOH (with or without CFW stain) similarly exhibits high sensitivity in detecting *Acanthamoeba* spp. (84–91%)^33 41^ and microsporidia (97%) [45]. GMS stain highlights fungal cell walls, which appear as black structures against a blue-green background, whereas LPCB stains fungal filaments in blue [46]. ZN stain or Kinyoun stain (modified ZN stain), which utilizes carbol fuchsin, is used to identify acid-fast bacteria such as *Mycobacterium* spp. or *Nocardia* spp. [34] Acid-fast organisms contain an additional component in the outermost part of their membrane, which consists of mycolic acid and large amount of lipids and waxes, rendering them not stainable by Gram stain. In addition, Mittal et al. [47] have recently reported the use of potassium iodide-sulfuric acid (IKI–H_2_SO_4_) in differentiating *Pythium insidiosum*, a pathogenic oomycete, from fungal filaments.

#### 2.1.3 Culture and sensitivity testing

Isolation of the organism on culture media remains the current gold standard in clinical practice and corroborating with stain results helps detect the causative organism definitively. In addition, it is the main method for determining the antimicrobial susceptibility and resistance profile of the organisms. Although broad-spectrum antibiotics are often administered in IK with good treatment response, culture results play a vital role in the management of non-responsive IK cases or those that are affected by polymicrobial infection or non-bacterial infections. Several studies have shown that culture and sensitivity results help guide and modify the antimicrobial treatment in around 5–15% of the IK cases, particularly in the severe cases [[48], [49], [50]].

Depending on the geographical location, study design, patient cohort and scraping methods, the culture positivity rate is reported to range between 24 and 77% [1,5,17,51,52]. Other factors such as older age, prior use of topical steroids, clinical severity, and presence of hypopyon have also been shown to influence the culture positivity rate [10,33]. Various culture techniques have been described, including direct plating on solid agars [5,19,53], indirect inoculation in liquid or transport media for subsequent culturing on solid agars [36,54], and direct and indirect inoculation methods used in combination [55]. In general, direct plating method is used when there is sufficient material for culturing on different agar plates whereas indirect inoculation in liquid phase media is used when the inoculum is small or when the patient has been on pre-treatment whereby the liquid medium dilutes the effect of the drug. McLeod et al. [36] demonstrated a comparable culture yield between Amies transport medium and direct plating. Similarly, Kaye et al. [56] found the results of direct plating to be comparable to indirect plating from brain heart infusion (BHI) medium. The advantage of using the indirect inoculation method lies in the technical simplicity as only one scrape is required for culturing, thereby saving time in a busy clinical setting. In addition, combined liquid and solid phase media have been shown to further improve the culture yield of bacterial and mixed IK [55].

A wide range of culture agars are available for culturing different types of microbes (Table 2) [54,[56], [57], [58], [59]]. After inoculating the samples in the media/agar, they are incubated and examined daily for a period of 1–2 weeks to assess growth of organisms. Organisms like *Acanthamoeba*, *Nocardia*, atypical *Mycobacteria* and fungi grow slowly and need prolonged incubation. In addition, growth of commensal organisms needs to be differentiated from the causative organism. Microbial growth is considered etiologically significant if: (a) the organism grows on two different media; (b) confluent (>10 colonies) growth is observed at the site of inoculation on solid media; (c) culture result is consistent with staining report; or (d) same organism is grown on repeat scrapes [34]. When clinically indicated, any potentially contaminated materials such as contact lenses, contact lens cases (with or without the cleaning solution), and loose corneal sutures should be sent for culture and sensitivity testing [8,60].

**Table 2:** A summary of commonly used culture media for various types of organisms.

Culture Media	Main Ingredients	B	F	A	N	MB
Blood agar	Peptone, tryptose, 5% sheep blood	Y	Y	–	Y	–
Chocolate agar	Similar to blood agar, but with lysed blood	Y^a^	Y	–	Y	–
Sabouraud agar	Dextrose, peptone	–	Y	–	Y	–
Potato dextrose agar (PDA)	Potato infusion, dextrose	–	Y	–	–	–
Non-nutrient agar with *E. coli* overlay	Peptone, yeast extract, beef extract, *E. coli*	–	–	Y	–	–
Lowenstein Jensen (LJ) medium	Potato flour, asparagine, malachite green, glycerol, potassium, magnesium	–	–	–	–	Y
Thioglycolate broth	Sodium thioglycolate, l-cystine, glucose, yeast extract, casein	Y^b^	–	–	–	–

B = Bacteria; F = Fungi; A = Acanthamoeba; N = Nocardia; MB = Mycobacteria.

a Neisseria and Hemophilus species.

b Differentiates obligate aerobes, obligate anaerobes and facultative anaerobes.

**Fig. 2:**
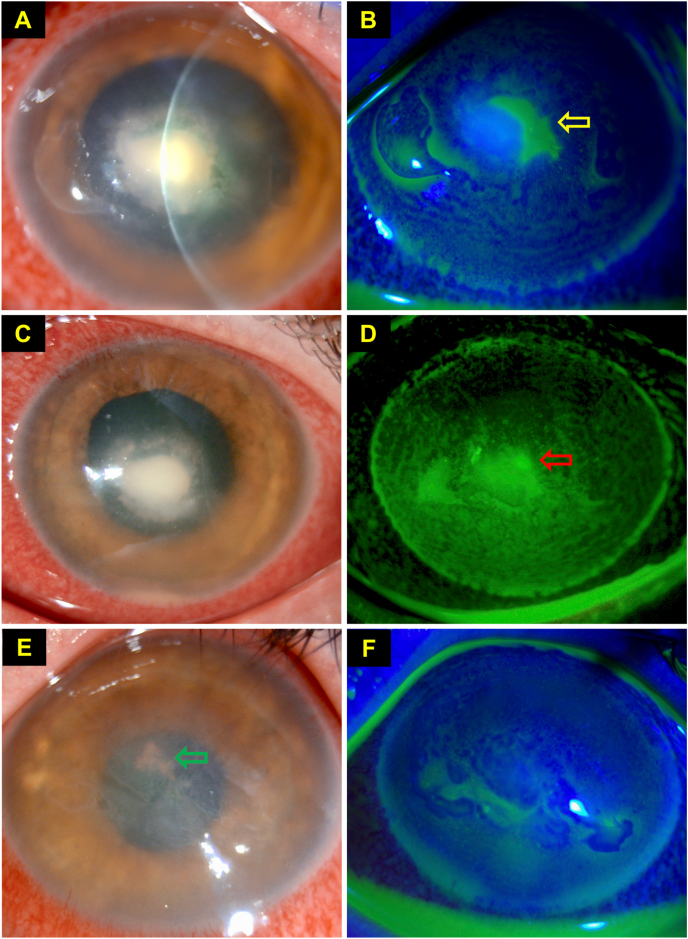
This figure highlights the diagnostic dilemma of infectious keratitis, even in the presence of positive microbiological culture result. This is a case of polymicrobial keratitis which was initially treated for a culture-proven *Staphylococcus aureus* keratitis with hourly topical antibiotics at day 0. **(A**–**B)** At day 4, slit-lamp photograph demonstrating a dense central infiltrate with healing epithelial defect (*yellow arrow*). **(C**–**D)** At day 5, slit-lamp photograph demonstrating continued improvement of the ulcer, with gradual contraction of the infiltrate and reduction of epithelial defect (*red* arrow). However, the deep-seated infiltrate failed to resolve after 10 days of intensive antibiotic treatment, raising the suspicion of co-existing fungal infection (confirmed on in vivo confocal microscopy). Topical antibiotics was then switched to topical voriconazole 1% hourly. **(E**–**F)** At day 24, complete healing of ulcer was achieved with intensive topical voriconazole drops. Deep central pigmented keratic precipitates (*green* arrow) and bullous keratopathy secondary to endothelial damage from IK were noted. (For interpretation of the references to colour in this figure legend, the reader is referred to the Web version of this article.)

Apart from diagnosis, culture of the organisms enables testing for the antibiotic susceptibility and resistance. The minimum inhibitory concentration (MIC), which is defined as the lowest concentration of an antimicrobial agent in preventing visible growth, can be determined using various methods, including the disc diffusion assays (using solid phase media) and broth macro- or micro-dilution method (using liquid phase media) [61,62]. Organisms are classified as either susceptible, intermediate, or resistant, dependent on the breakpoints set by the Clinical and Laboratory Standard Institute (CLSI) and European Committee on Antimicrobial Susceptibility Testing (EUCAST) guidelines. However, treatment of IK based on antimicrobial susceptibility results has been a matter of debate. The antimicrobial susceptibility is determined by the systemic breakpoints instead of ophthalmic breakpoints. As such, the anticipated treatment response of the organism is based on the serum concentration of the antimicrobial drug rather than the concentration achieved by topical administration, which consequently can be misleading [27,63]. A drug to which the organism is reportedly resistant may prove to be effective in resolving the IK owing to higher concentrations achieved by fortification and frequent instillations [63,64]. In contrast, IK has also shown to worsen while on the drug they were reportedly sensitive to [48]. It is therefore important to interpret the microbiological and antimicrobial susceptibility results alongside with the clinical progress. For instance, the patient should remain on the same antimicrobial treatment regimen if there is a positive clinical response, even if the microorganism is found to be resistant to the drug. On the other hand, if the IK worsens despite the identified organism is being reported as susceptible to the ongoing antimicrobial agent, it should raise the clinical suspicion of polymicrobial infection such as mixed bacterial and fungal infection (Fig. 2A–F) [1].

#### 2.1.4 Corneal biopsy

Corneal biopsy may serve as a useful technique for unveiling the causative organism in progressive or refractory IK, particularly when the initial culture result is negative [[65], [66], [67]]. Compared to corneal scraping, it has the advantage of obtaining the samples from deeper layers of cornea and/or debulking the infection, with an overall diagnostic yield of approximately 39–82% [[65], [66], [67]].

Alexandrakis et al. [65] demonstrated a high diagnostic yield of corneal biopsy in 33 cases of IK that were not responsive to antimicrobial therapy, with organisms being isolated in 82% of cases. More importantly, the identification of organisms had guided and altered the antimicrobial treatment in 89% of cases which were culture negative on corneal scrapes. Younger et al. [66] reported a higher rate of microorganism identification on histopathologic examination than culture of the corneal biopsies (40% vs. 19%), suggesting that both examinations should be performed to increase the overall yield.

While corneal biopsy serves as a useful diagnostic procedure, it is important to note that this procedure is not without risk. Inadvertent corneal perforation associated with biopsy has been reported [66], especially if there is pre-existing corneal thinning, melting and necrosis (which may give a false impression of a thick and swollen cornea). As all antimicrobial treatments are usually discontinued prior to corneal biopsy, close monitoring for any sudden clinical deterioration is warranted.

In our practice, a corneal biopsy is indicated when IK progresses despite intensive antimicrobial therapy, especially in the absence of positive microbiological results. All antimicrobial treatments are usually withheld for 24–48 h before corneal biopsies are performed to increase the culture yield. The procedure is carried out in either minor or main operating theatre, depending on severity of corneal thinning and risk of perforation. A 2- or 3-mm round, sterile dermatologic trephine is used to mark and advance to the anterior stroma of the infected site, followed by a superficial lamellar keratectomy using a crescent blade. At least two corneal biopsies are obtained, with one sample being sent for microbiological investigations such as microscopy, staining and culture and PCR, and the other sample being sent for histopathological examination with emphasis on examination for Acanthamoeba and fungal infections. Intensive antimicrobial treatment is then restarted while awaiting the culture and histopathological results. Other technique such as lamellar flap corneal biopsy for gaining access to deep infiltrate has also been described [68].

### 2.2 Molecular diagnostics and other corneal sampling technique

#### 2.2.1 Polymerase chain reaction

Polymerase chain reaction (PCR) represents one of the most significant breakthroughs in the realm of biomedical science [69]. It is a rapid and highly sensitive enzymatic assay that enables amplification of a targeted deoxyribonucleic acid (DNA) fragment within a DNA sample. A number of key ingredients are required for the assay, including template DNA, predetermined primers (which are short, single-stranded DNA sequences that complement the targeted DNA), nucleotides (or deoxynucleic triphosphates; dNTPs), and DNA polymerase (which is a thermostable enzyme that synthesizes new strands of DNA complementary to the targeted DNA sequence). The process involves repeated cycles of denaturation, annealing, elongation and replication of the targeted DNA sequence, which generates billions of copies of the targeted DNA at the end of the process (usually within 1–2 h).

As such, only a small amount of DNA is required to yield a positive result, rendering PCR a highly sensitive test. In addition, PCR has been explored and utilized in profiling antimicrobial susceptibility and resistance [70]. Real-time or quantitative PCR, a subtype of PCR, allows analysis of the number of targeted DNA in real-time via monitoring of the level of fluorescence [71]. Based on the quantity of the targeted microbial DNA in the presence and absence of antimicrobial agents (and comparing with control groups), antimicrobial susceptibility can be rapidly determined, potentially serving as a novel supplementary method to the conventional solid and liquid phase susceptibility testing [72,73]. Other PCR-based technique such as nested PCR (uses two sets of primers and two successive PCR reactions to improve the detection sensitivity and specificity), multiplex PCR (amplifies different DNA sequences simultaneously), and reverse transcriptase PCR (amplifies complementary DNA that are derived from a RNA sample) [70].

In view of its superior diagnostic performance, rapid turnaround diagnostic time and versatility, PCR has been increasingly used to improve the diagnosis of a wide range of infectious diseases [70]. Within the context of IK, PCR has been applied to the full range of microorganisms, including bacteria, fungi, Acanthamoeba and viruses. A summary of the recent literature (i.e. studies published after year 2010) on the use of PCR in IK is provided in Table 3 [[74], [75], [76], [77], [78], [79], [80], [81], [82], [83], [84], [85], [86], [87], [88], [89], [90], [91], [92], [93]].

**Table 3:** A summary of the recent evidence on the use of polymerase chain reaction (PCR) in the diagnosis of a range of infectious keratitis.

**Year**	**Authors**	**Sample size**	**Technique**	**Target region**	**Sen (%)**	**Spec (%)**	**Comparison (if any)**
*Bacterial keratitis*							
2020	KrishnanNair et al. [74]	100	Multiplex qPCR	Species specific DNA region	100	100	–
2019	Wagner et al. [75]	499	qPCR	16S rDNA	94.5	100	–
2019	Shimizu et al. [76]	118	qPCR	16S rDNA	63.6	67.5	Culture = 51.8 (Sen), 77.2 (Spec); Smear = 63.1 (Sen), 89.8 (Spec)
2017	Fang et al. [77]	61	DBH	16S rDNA	81.3–93.8	71.1–100	–
2015	Panda et al. [78]	122	qPCR	16S rDNA	89	87	Smear = 45.3 (Sen), 92.8 (Spec)
*Fungal keratitis*							
2020	Kulandai et al. [79]	42	qPCR	18S RNA & ITS	98.2	100	–
2020	Ren et al. [80] a	35	qPCR	ITS	74.3	–	IVCM = 77.1; Smear = 77.1; Culture = 71.4
2018	Wagner et al. [81]	233	qPCR (18S rDNA), semi-nested qPCR (ITS)	18S rDNA, ITS	97.8 (18S) 86.7 (ITS)	100 (18S) 100 (ITS)	–
2015	Haghani et al. [82]	40	Semi-nested PCR	ITS	57.1	78.7	Smear = 28.5–42.0 (Sen), 78.7–94.0 (Spec)
2014	Zhao et al. [83]	80	Touchdown PCR	ITS	98.0	81.8	Culture = 47.1 (Sen), 100 (Spec)
*Acanthamoeba keratitis*							
2018	Goh et al. [84]	25	qPCR	18S rDNA	71.0	100	Culture = 33.3 (Sen), 100 (Spec); IVCM = 100 (Sen), 100 (Spec)
2017	Karsenti et al. [85]	107	qPCR	18S rDNA	100	96.0	–
2017	Mewara et al. [86]	42	PCR, LAMP	18S rDNA	100 (PCR, LAMP)	100 (PCR, LAMP)	Smear = 60 (Sen), 100 (Spec); Culture = 100 (Sen), 100 (Spec)
2016	Huang et al. [87]	20	DBH	18S rDNA	87.5	100	–
2015	Kowalski et al. [88]	125	PCR	18S rDNA	85.7	100	Culture = 81.0 (Sen), 100 (Spec)
*Viral keratitis*							
2019	Brunner et al. [89] a	110	qPCR	HSV DNA	25.5–43.8	–	–
2019	Guda et al. [90]	50	Multiplex qPCR	HSV and VZV DNA	100	28	–
2018	Inata et al. [91] a	38	qPCR	VZV DNA	84.2	–	–
2016	Ma et al. [92] a	30	qPCR	HSV DNA	46.4	–	–
2016	Kuo et al. [93] b	33	Multiplex DBH	HSV DNA	93.3	100	–

Sen = Sensitivity; Spec = Specificity; qPCR = Quantitative or real-time PCR; DBH = Dot-blot hybridization; rDNA = ribosomal DNA; ITS = Internal transcribed sequence; LAMP = Loop-mediated isothermal amplification; HSV = Herpes simplex virus; VZV = Varicella zoster virus.

a These studies reported the detection rate.

b This study examined both Acanthamoeba keratitis and herpes simplex keratitis.

Ribosomes and ribosomal RNA (rRNA), which are regulated by the respective rDNA, are essential to organism survival and are evolutionarily maintained during the natural selection. Mutation differences evolved over time have resulted in the disparity in rRNA of the three domains of life, namely the Bacteria, Archaea and Eukarya [94]. 16S rRNA (or rDNA), the core component of the 30S small subunit, is highly conserved by the prokaryotes such as bacteria and archaea, and hence is often used as a PCR diagnostic target for bacterial keratitis. Based on the recent evidence, the overall sensitivity and specificity of PCR in diagnosing bacterial keratitis are 64–100% and 68–100%, respectively [[74], [75], [76], [77], [78]]. Shimizu et al. [76] reported that 16S rDNA PCR exhibited a similar diagnostic performance to conventional microbiological method (e.g. smear and culture) in bacterial keratitis and the diagnostic efficacy could be improved when these methods are used in combination.

18S rRNA – the main component of 40S small subunit – is highly conserved by eukaryotes, including fungi and Acanthamoeba, and is therefore used as a target for PCR-based diagnosis [94]. In addition, internal transcribed spacer (ITS), which is a spacer DNA (a region of non-coding DNA between genes) that locates between small and large subunit rRNA, has also been used as a target for diagnosing fungal keratitis [7,95]. Two ITS are present In eukaryotes, namely ITS1 (which is flanked by 18s rRNA and 5.8S rRNA) and ITS2 (which is flanked by 5.8S rRNA and 28S rRNA). Compared to 18S rRNA, ITS has been shown to exhibit higher species-level resolution in fungi, serving as a primary fungal barcode marker [95]. The overall sensitivity and specificity of PCR in diagnosing fungal keratitis are 57–91% and 79%, respectively [[79], [80], [81], [82], [83]]. Zhao et al. [83] demonstrated that PCR utilizing ITS1 and ITS4 primers, which amplify ITS1 and ITS2 sequences respectively, had a significantly higher detection rate for fungal keratitis compared to conventional culture method (85% vs. 35%). In addition, the time taken to reach the diagnosis was substantially faster than conventional culture (3 h vs. few days). Another study found that PCR based on 18S rDNA had a better detection rate of fungi than ITS-based PCR and culture [81], suggesting that both 18S rDNA and ITS-based PCR are useful in aiding the diagnosis of fungal keratitis.

PCR has also been employed in diagnosing Acanthamoeba keratitis, with an overall sensitivity and specificity of 71–100% and 96–100%, respectively [84,85,87,88,96]. Loop-mediated isothermal amplification (LAMP), a newly developed PCR-based technique, has also been explored to diagnose Acanthamoeba keratitis, with comparable efficacy with conventional PCR method but less time-consuming as it is performed under isothermal conditions [86,97]. PCR has also been commonly used in diagnosing and distinguishing different types of viral keratitis, particularly herpes simplex keratitis (HSK) and herpes/varicella zoster keratitis (HZK) [89,98,99].

Despite its superior diagnostic value in infectious diseases, PCR has several inherent weaknesses that are worth noting. First, PCR only amplifies a targeted DNA sequence based on a specific primer (i.e. highly specific), therefore only the targeted organisms will be examined and analyzed. Second, PCR can amplify not only the pathogens but also the normal flora from “background contamination”, potentially obfuscating the clinical findings with resultant false positivity [76,100]. In addition, studies have shown that microbial DNA can be detected despite following successful antimicrobial treatment, complicating the interpretation of PCR findings [70]. Cost and accessibility represents another barrier to the adoption of PCR in clinical practice as it is associated with a considerably higher cost than conventional culturing and is only available in some specialized units.

#### 2.2.2 Mass spectrometry

Mass spectrometry (MS) serves as another novel molecular diagnostic tool in the field of infectious diseases. It provides accurate quantitative analysis of the biological samples, including microorganisms, in mass-to-charge ratio (*m*/*z*). Depending on the type of ionization technique, electrospray ionization (ESI) and matrix-assisted laser desorption/ionization (MALDI), with or without time of flight (TOF), MS are the two most commonly used approaches for identifying microorganisms [101].

In ophthalmology, MS has been used to identify and characterize the organisms of IK in some units [102,103]. Based on the distinctive mass spectral fingerprints, MS is able to accurately and rapidly resolve the pathogens to species or even subspecies level [[103], [104], [105]]. In addition, this technique is particularly useful for identifying rare organisms, particularly when the morphological characteristics of the particular organism are not well known or defined. Ting et al. [106] previously demonstrated the utility of MALDI-TOF-MS in identifying a rare organism, *Arthrographis kalrae*, where the initial culture result showed a nonspecific appearance of “mold”.

#### 2.2.3 Impression cytology

Impression cytology is another useful technique that can obtain cellular or microbial samples from the ocular surface. This technique was first described by Egbert et al. [107] in 1977 as a simple way of obtaining conjunctival biopsies. After topical anesthesia, superficial layers of the ocular surface, including the corneal and/or conjunctival epithelium, are obtained via the application of a cellulose acetate filter paper (or nylon paper in some practice), for which further histological, immunohistochemical and molecular analysis can be performed [108,109].

Within the context of IK, impression cytology has demonstrated its diagnostic value in cases of Acanthamoeba keratitis [[110], [111], [112]] and viral keratitis/keratoconjunctivitis [113,114]. Florakis et al. [111] first reported the use of impression cytology in culturing and diagnosing Acanthamoeba keratitis. Sawada et al. [112] subsequently utilized impression cytology to diagnose 3 patients with Acanthamoeba keratitis via histopathological examination with modified Papanicolaou staining [112]. This technique was able to quickly reveal the presence of Acanthamoeba cysts (stained in dark-bluish colour) and trophozoites (stained in pink or light purple) compared to culture. However, as impression cytology only captures the superficial cells (or cells at a slightly deeper location via repeat applications at the same site) [108], it is less useful for diagnosing deep-seated Acanthamoeba infection. Alcohol delamination of the corneal epithelium has also been described to achieve the same diagnostic purpose for Acanthamoeba keratitis [115]. In addition, impression cytology has been used to rapidly diagnose viral keratitis secondary to herpesviruses and adenovirus using targeted monoclonal antibodies and immunofluorescence techniques, with good specificity and sensitivity [114,116].

### 2.3 Imaging modalities

In current clinical practice, evaluation of the clinical morphology and severity of IK are largely achieved through slit-lamp microscopy. However, several adjuvant imaging modalities have shown their clinical utility in diagnosing and monitoring the progress of IK in the past decade. This section briefly summarizes the potential utility, strengths and limitations of each modality.

#### 2.3.1 Anterior segment optical coherence tomography

**Fig. 3:**
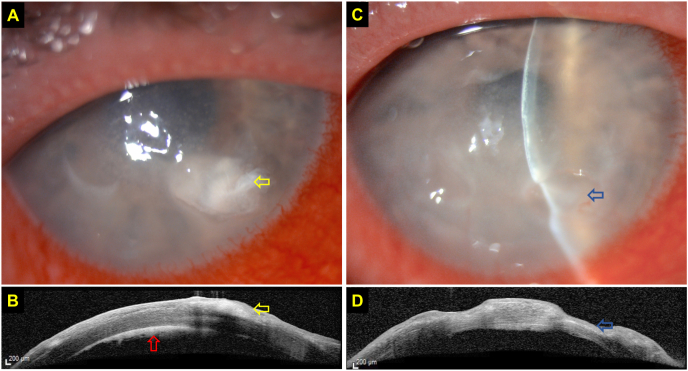
This figure highlights the clinical value of anterior segment optical coherence tomography (AS-OCT) in assessing and monitoring infectious keratitis. **(A)** Slit-lamp photography demonstrating a case of culture-negative, presumed right fungal keratitis with a moderate-size infiltrate (*yellow arrow*) at the inferonasal aspect of the cornea. **(B)** AS-OCT clearly delineates the margin and depth of the infiltrate (*yellow arrow*), located at the anterior ½ of the stroma, and highlights the presence of a retrocorneal membrane (*red arrow*), highly suggestive of fungal keratitis. **(C**–**D)** AS-OCT demonstrating a significant reduction in the corneal infiltrate with moderate corneal thinning (*blue arrows*) after one month of intensive topical antifungal treatment. (For interpretation of the references to colour in this figure legend, the reader is referred to the Web version of this article.)

Anterior segment OCT (AS-OCT) has been used to examine a wide array of corneal pathologies. The main advantages of AS-OCT over slit-lamp microscopy lie in its ability to accurately determine and delineate the depth and extent of corneal ulceration, infiltrates and haze, which can be used to characterize, quantify and monitor the progress of various corneal pathologies, including superficial and deep-seated IK [[117], [118], [119], [120], [121], [122], [123]]. This is particularly useful in IK where significant necrotic tissues or infiltrate could obscure the view of underlying tissues (Fig. 3A–D). AS-OCT can also be used to objectively measure corneal thickness, determine the risk of corneal perforation, and predict the treatment response of IK following therapeutic corneal cross-linking (PACK-CXL), which has been shown to be less effective for deep-seated IK [[124], [125], [126], [127]]. AS-OCT can also be employed to highlight corneal interface pathologies such as interface IK following lamellar keratoplasty or post-LASIK epithelial ingrowth, which appears as a hyper-reflective band at the graft-host interface and flap-host interface, respectively [128,129], and valvular and direct non-traumatic corneal perforations associated with IK [130].

In addition, several characteristic features of IK, including fungal, Acanthamoeba and viral keratitis, have been observed on AS-OCT [123,[131], [132], [133], [134], [135]]. Soliman et al. [131] examined the AS-OCT images in 20 patients with bacterial or fungal keratitis and observed two unique features that were suggestive of fungal infection, namely localized and diffuse stromal cystic spaces caused by stromal necrosis. Characteristic features of Acanthamoeba keratitis such as radial keratoneuritis or perineural infiltrates may also appear as hyper-reflective bands in the corneal stroma in varying width (20–200 μm) and depth (subepithelial to mid-stroma) [132,133].

#### 2.3.2 In vivo confocal microscopy

In vivo confocal microscopy (IVCM) serves as another valuable imaging modality that enables non-invasive, high-resolution, in vivo evaluation of corneal structures and pathologies at a cellular and sub-cellular level [[136], [137], [138]]. It has been considered as a non-invasive method of “in vivo corneal biopsy”. Various types of IVCM have so far been developed and applied to clinical practice for imaging the anterior segment, particularly the cornea. Among them, laser scanning IVCM [Heidelberg Retinal Tomograph (HRT), Heidelberg Engineering, GmBH, Germany] [138] combined with a specially designed, mountable objective system, named Rostock Corneal Module (RCM), has been the preferred choice of IVCM in our practice as well as many others [139]. HRT-RCM system is able to produce higher quality images, with a lateral resolution of 1 μm, axial resolution of 7.6 μm and 400x magnification [[140], [141], [142]].

A number of previous reviews have summarized the use of IVCM in ophthalmology [136,138,143], and this section aims to mainly recapitulate the clinical utility of IVCM in IK. To date, IVCM has mainly been employed in the assessment of fungal and Acanthamoeba keratitis. This is because the current axial resolution of IVCM is limited to 5–7 μm and is not sufficient to resolve bacteria (usually <5 μm) or viruses (in nanometres) [141,144]. Diagnosis of fungal keratitis using standard microbiology investigations such as staining and culture has been challenged by the variable yield rate (40–99%) and slow turnaround time (∼25% cases take up to 2 weeks incubation period to yield a positive result) [35,36,40,145]. Therefore, any additional investigation that could improve the time to diagnosis and positive yield in fungal keratitis would be clinically valuable.

According to the literature, the overall sensitivity and specificity of IVCM in detecting fungal pathogens, particularly filamentous fungi, is estimated at 66.7%–85.7% and 81.4–100%, respectively (Table 4) [84,[146], [147], [148], [149], [150]]. *Aspergillus* spp. and *Fusarium* spp. are two of the most common fungi implicated in fungal keratitis [151]. These filamentous fungi appear as high-contrast, hyper-reflective lines resembling hyphae, with 45- or 90-degree branching patterns, on the IVCM whereas *Candida* spp. (yeast-like fungi) appear as elongated, hyper-reflective particles resembling pseudofilaments [147,152]. While some studies have demonstrated the potential ability of IVCM to sub-classify filamentous fungi (e.g. *Aspergillus* spp. vs. *Fusarium* spp.) based on the branching patterns, a recent large prospective study had failed to support this claim [153].

**Table 4:** A summary of the diagnostic performance of in vivo confocal microscopy (IVCM) on infectious keratitis (IK).

Author	Year	IVCM system	Sample size	Sensitivity	Specificity
Wang et al. [145] a	2019	HRT3/RCM	49	66.7 (F); 91.7 (A); 66.7 (B); 100 (V)	100 (F); 100 (A); 89.2 (B); 93.2 (V)
Goh et al.84	2018	HRT2/RCM	15 (A); 11 normal	100 (A)	100 (A)
Chidambaram et al. [146]	2016	HRT3/RCM	176 (F) 17 (A)	85.7 (F); 88.2 (A)	81.4 (F); 98.1 (A)
Vaddavalli et al. [147]	2011	Nidek ConfoScan 3.0	93 (F); 10 (A)	89.2 (F); 80.0 (A)	92.7 (F); 100 (A)
Hau et al. [148] b	2010	HRT2/RCM	15 (F); 26 (A); 21 (B)	27.9–55.8	42.1–84.2
Kanavi et al. [149]	2007	Nidek ConfoScan 3.0	16 (F); 15 (A)	94.0 (F); 100 (A)	78.0 (F); 84.0 (A)

HRT/RCM = Heidelberg Retinal Tomography (version 2 or 3) with Rostock Corneal Module.

F = Fungi; A = Acanthamoeba; B = Bacteria; V = Viruses.

a This study included all types of IK, including bacterial, fungal, Acanthamoeba, viral and polymicrobial infection.

b This study included bacterial, fungal and Acanthamoeba infection.

**Fig. 4:**
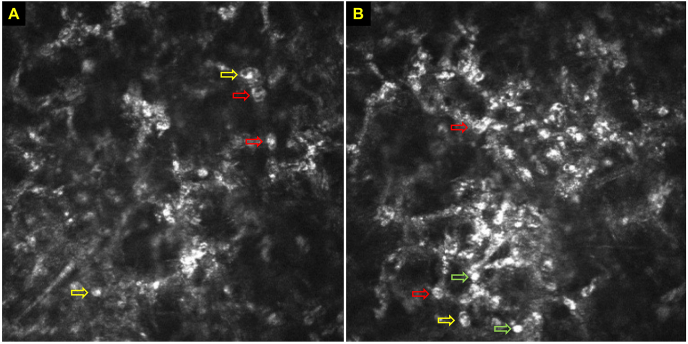
**(A**–**B).** Various characteristic features of Acanthamoeba cysts on in vivo confocal microscopy (IVCM), including double-wall cysts (*red arrows*), signet rings *(yellow arrows*), and bright spots (*green* arrows). (For interpretation of the references to colour in this figure legend, the reader is referred to the Web version of this article.)

IVCM has proven to be a valuable addition to the diagnostic armamentarium of Acanthamoeba keratitis. *Acanthamoeba* spp. may present in various morphological appearances on IVCM, including: (a) double-walled cysts (the dormant form) of ∼15–30 μm in diameter located at epithelium and/or stroma IVCM, which is the most widely reported IVCM feature of Acanthamoeba keratitis; (b) trophozoites (the active form) of 25–40 μm appearing as hyper-reflective structures, though not easily distinguishable from other hyper-reflective changes in infected corneas; (c) bright spots; (d) signet rings; and (e) perineural infiltrates, a pathognomonic feature of Acanthamoeba keratitis, appearing as highly reflective patchy lesions with surrounding hyper-reflective spindle-shaped materials (Fig. 4A–B) [141,154,155]. Interestingly, clusters of cysts were observed following the topical steroids, which has been associated with poor prognosis [156,157]. While the underlying reason is unclear, this sign might resemble the mechanism of “biofilm formation” observed in other types of infection [158], though further investigation is required to support this. Overall, IVCM exhibits a superior diagnostic performance in detecting Acanthamoeba keratitis, with an overall sensitivity and specificity of 80.0–100% and 84.0–100%, respectively (Table 3) [84,[146], [147], [148],^150^].

In clinical setting, IVCM is particularly useful in IK cases when the culture result is negative and the infection is deep-seated, either due to the natural clinical course of some fungal keratitis [159] or development of interface IK following corneal surgeries, which limits the access of standard microbiological investigations [160]. Recently, we reported the use of IVCM in a challenging case of culture-negative interface IK following Descemet's stripping automated endothelial keratoplasty [128]. The rapid detection of possible hyphae on IVCM had led to timely initiation of anti-fungal treatment, culminating in a favorable clinical outcome.

## 3 Recommendations

As there is currently no one-size-fits-all diagnostic approach for IK, we recommend that conventional microbiological investigations such as corneal scraping for microscopy, culture and sensitivity should be considered and performed in all patients with: (1) ulcer >2 mm; (2) sight-threatening infection; and/or (3) atypical infection [10]. The corneal sampling technique needs to be standardized and optimized according to the local guideline of the institutions, with close collaboration between the local ophthalmology and microbiology team, whilst being cognizant of the potential strengths and limitations of the chosen method. Where resources and facilities are available, combined conventional microbiology and molecular diagnostics could further enhance the diagnostic yield and accuracy of IK. Serial imaging of IK, utilizing a combination of slit-lamp photographs and AS-OCT, could provide objective quantification of the baseline severity and progression of the IK during the treatment period. It is also noteworthy to mention that, while these imaging modalities are for visualizing and monitoring IK, none of the imaging findings are pathognomonic and should always be interpreted along with the clinical findings and microbiological results. IVCM serves as a powerful adjuvant tool for IK, particularly in culture-negative cases where Acanthamoeba or fungal infection is suspected. However, the use and interpretation of IVCM is highly subject to operator's experience [149].

## 4 Future directions

### 4.1 Clinical metagenomic next generation sequencing

Next generation sequencing (NGS) is a term used to describe a number of different high-throughput sequencing techniques that enable rapid, massive parallel sequencing of DNA and RNA [161]. This culture independent technology is also referred to high-throughput sequencing and massive parallel sequencing. Unlike the traditional Sanger sequencing method which sequences a single DNA fragment at a time, NGS can sequence all genomic contents of a given sample within a very short turnaround time, thereby providing a comprehensive examination of all DNA or RNA within the sample. In view of these advantages, NGS has been gaining traction in the field of infectious diseases in the recent years, including for the diagnosis of IK and assessment of ocular surface microbiome in healthy and diseased states [[162], [163], [164]].

#### 4.1.1 Types of NGS approaches/platforms

In principle, NGS approaches for the diagnosis of infectious diseases can be divided into two types, namely PCR-targeted amplicon sequencing and shotgun metagenomic deep sequencing. Targeted amplicon sequencing uses PCR primers to amplify regions of highly conserved microbial DNA. The most commonly amplified target genes are 16S rDNA, 18S rDNA, and ITS genes. In general, amplicon sequencing is used when microbial analysis focuses on bacterial (16s rDNA) and fungal (18s rDNA and ITS) identification. Shotgun metagenomic sequencing, also called metagenomic deep sequencing (MDS), is truly hypothesis-free and analyses all DNA or RNA in a sample, with RNA sequencing requiring special processing. Metagenomic sequencing allows for the identification of all microbial RNA and DNA including viruses and parasites and allows for the discovery of unexpected organisms [[162], [163], [164]]. Amplicon sequencing is targeted, somewhat less expensive and faster. Metagenomic sequencing carries the advantage of being more unbiased; however, is more expensive, and has a longer processing time. All types of NGS involve four main steps, including library preparation, clonal amplification, massive parallel sequencing, and data analysis [161].

#### 4.1.2 Clinical techniques and considerations

Collection and processing of clinical samples for NGS require special considerations. Because this technology is so sensitive to microbial detection, it is also prone to a high false positive rate because of microbial contamination. Contamination can occur at every step from collection through processing and all efforts should be made to minimize environmental and laboratory contamination [165]. Immediately after the swab is obtained, the sample is placed into DNA/RNA stabilization solution, which preserves the genetic material in the sample as well as inactivates the infectivity of all pathogens. The sample is then placed promptly in a −20 °C freezer and transferred to −80 °C when possible [166]. Freeze thaw cycles are to be minimized as they can degrade the genetic material. RNA, in particular, is very susceptible to degradation at room temperature. When the sample is ready for sequence analysis, it is processed through both a “wet lab” and “dry lab”. In the laboratory “wet lab”, DNA is extracted, or RNA is extracted and converted to cDNA. For amplicon sequencing, the next step is targeted PCR amplification. Here, PCR primers are directed toward known highly conserved genes. These genes also contain variable regions that are simultaneously amplified by this process. Sequence analysis of these variable regions allows for bacterial and fungal species identification. For deep sequencing, DNA is randomly sheared into small fragments. Techniques for depleting human DNA are applied in order to enrich for microbial DNA. Universal primers are attached to the remaining fragments allowing for amplification using PCR. For both sequencing techniques, the next steps are library preparation and DNA sequencing. Once the sequence data are available, the “dry lab”, meaning computational bioinformatic algorithms, decode and analyze the output. Using several different bioinformatic algorithms, still being refined, the DNA identified as host and commensal organisms are subtracted and the presumed causative pathogen is identified by matching sequences to one of several large known pathogen databases, such as the National Center for Biotechnology Information (NCBI) GenBank nucleotide database.

#### 4.1.3 Clinical applications and limitations in infectious keratitis

To date, only a few studies have evaluated the potential utility of MDS for diagnosing IK [[166], [167], [168], [169]]. Seitzman et al. [166] compared the diagnostic efficacy of MDS with conventional culture and viral PCR in patients with IK and demonstrated that MDS was able to unveil all range of causative microorganisms (including bacteria, fungi, Acanthamoeba and herpes simplex virus) in one single assay. The versatility of MDS in diagnosing all types of IK, including culture-negative cases, has been demonstrated in other studies [[167], [168], [169], [170]].

However, it is noteworthy to highlight that NGS has several clinical limitations. Low-biomass clinical samples, such as with a typical corneal inoculum, is particularly prone to background contamination [171]. As NGS analyzes all the RNA or DNA fragments within a sample, amplification of background contamination (acquired in the clinical or laboratory space) or even amplification of normal ocular surface flora, can yield false positive results. There are several potential bioinformatic strategies to circumvent these limitations. These include subtraction analysis from air samples, from water control samples present on the same sequencing run and “normal flora control” samples from the unaffected, contralateral eye.

However, bilateral infections and secondary infections attributed to organisms commonly thought of as commensal such as Staphylococcus, Streptococcus or Corynebacteria require special bioinformatic considerations. In these instances, comparative analysis of absolute number of reads, rather than sequence subtraction can be considered. Continued development, refinement, and standardization of bioinformatic tools, techniques, resources, and databases will allow for further increases in the specificity of deep sequencing as a tool for infectious disease diagnosis [166]. The processing time from sampling to result may take several days and sequencing costs more than current conventional microbiological methods. Targeted amplicon sequencing is often less than $100 per sample. MDS, depending on the depth of sequencing reads, ranges between $200–500 per swab from a single patient [166].

### 4.2 Digital health

The simultaneous evolution of artificial intelligence (AI) technologies, particularly with deep learning (DL) and machine learning (ML), tele-medicine, and internet-of-things (IoT) has rapidly ushered in the era of digital health in recent years [172,173]. These technologies have demonstrated their potential in improving the workflow efficiency and addressing the ever-increasing workload in healthcare services [174,175]. The need for digital health is further amplified by the recent COVID-19 pandemic, which has significantly affected ophthalmic patients and the ophthalmic services [173,[176], [177], [178]].

So far, AI has shown promises in a broad range of ophthalmic conditions, including diabetic retinopathy, glaucoma, retinopathy of prematurity, cataract, and corneal diseases, amongst others [[179], [180], [181], [182]]. Kuo et al. [183] successfully developed a DL-based corneal photograph model to automatically detect fungal keratitis, with a mean accuracy of ∼70% (which was comparable to ophthalmologists who were not corneal specialist). Automated detection of fungal hyphae using DL-based IVCM photographs have also been described to improve the diagnosis of fungal keratitis [184,185]. More recently, Li et al. [53] demonstrated the potential of using a DL-based algorithm with slit-lamp photographs to accurately diagnosing a range of anterior segment diseases, including IK, pterygium and conjunctivitis, and cataract., and making automated recommendation for the subsequent treatment plan.

In addition, tele-medicine has been explored and implemented to assess and diagnose various corneal diseases, including IK [[186], [187], [188]]. For instance, Maamari et al. [186] reported the use of a tele-medicine platform in diagnosing corneal epithelial defect and IK based on white and fluorescein images captured by mobile phone. The diagnostic performance was found to be superior with a sensitivity and specificity of >80% and >90%, respectively, and was comparable to the accuracy of the on-site ophthalmologists.

## 5 Conclusions

IK represents a common and persistent burden to human health in both developed and developing countries. Timely and accurate diagnosis represents the cornerstone of the management of IK. Conventional microbiological testing is currently considered the gold standard for diagnosing IK, albeit challenged with a number of inherent limitations such as variably low sensitivity and long turnaround time. The recent advancement in imaging techniques, molecular diagnostics and AI technologies are likely to refine and shape the diagnostic landscape of IK in the near future. However, further work is required to examine and validate the clinical performance of these emerging technologies in the real-world setting. Furthermore, as IK is most prevalent in under-resourced regions, the accessibility, costs and cost-effectiveness of these technologies need to be further improved.

## 6 Methods of literature review

Relevant electronic databases, including MEDLINE OVID (January 1950–July 2021) and EMBASE OVID (January 1980 to July 2021), were searched for relevant articles related to the diagnosis of infectious keratitis. Keywords such as “infectious keratitis”, “microbial keratitis”, “corneal ulcer”, “diagnosis”, “corneal scrape”, “smear”, “culture”, “imaging”, “polymerase chain reaction”, “mass spectrometry”, “next-generation sequencing, “artificial intelligence” and “tele-medicine” were used. Only articles published in English were included. Bibliographies of included articles were manually screened to identify further relevant studies.

## Funding / Support

D.S.J.T. acknowledges support from the 10.13039/501100000265Medical Research Council / Fight for Sight (FFS) Clinical Research Fellowship (MR/T001674/1) and the FFS / John Lee, Royal College of Ophthalmologists Primer Fellowship (24CO4).
